# Bepostastine besylate

**DOI:** 10.1107/S2414314626004244

**Published:** 2026-04-29

**Authors:** Jacob K. Salazar, James A. Kaduk, Anja Dosen, Thomas N. Blanton

**Affiliations:** a North Central College, Department of Chemistry 131 S Loomis St Naperville IL 60540 USA; b North Central College, Department of Physics 131 S Loomis St Naperville IL 60540 USA; c Illinois Institute of Technology, Department of Chemistry, 3101 S. Dearborn St., Chicago, IL 60616, USA; d ICDD, 12 Campus Blvd, Newtown Square, PA 19073-3273, USA; e ICDD, 12 Campus Blvd., Newtown Square, PA 19073-3273, USA; Purdue University, USA

**Keywords:** powder diffraction, bepotastine, bepreve, Rietveld refinement, density functional theory, DFT

## Abstract

The crystal structure of bepotastine besylate {systematic name: 1-(3-carb­oxy­prop­yl)-4-[(4-chloro­phen­yl)(pyridin-2-yl)meth­oxy]piperidin-1-ium benzene­sulfonate, C_21_H_26_ClN_2_O_3_^+^·C_6_H_5_O_3_S^−^) was refined using synchrotron X-ray powder diffraction data, and optimized using density functional theory techniques. Bepotastine besylate crystallizes in the space group *P*1, with *a* = 8.0153 (6), *b* = 9.8211 (5), *c* = 10.2345 (10) Å, α = 88.164 (2), β = 68.962 (2), γ = 65.8917 (8)°, *V* = 680.149 (1) Å^3^ and *Z* = 1 at 298 K. N—H⋯O and O—H⋯O hy­dro­gen bonds link the cations and anions into a chain extending parallel to the *c* axis, with a graph set *C*_2_^2^(11).

## Abstract 2

The crystal structure of bepotastine besylate was refined using synchrotron X-ray powder diffraction data and optimized using density functional theory techniques.

## Graphical abstract

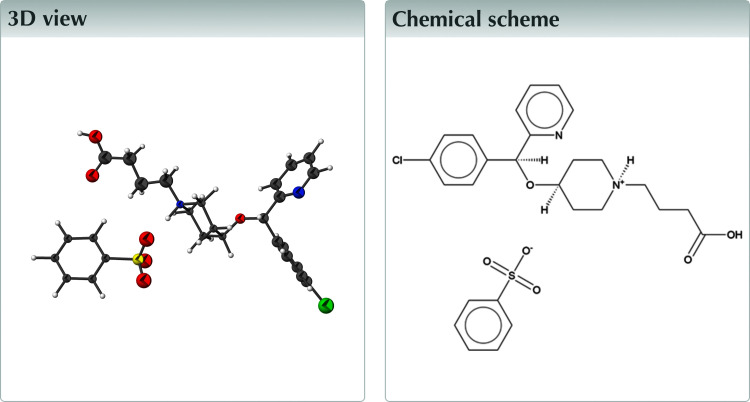

## Structure description

**Figure 1:**
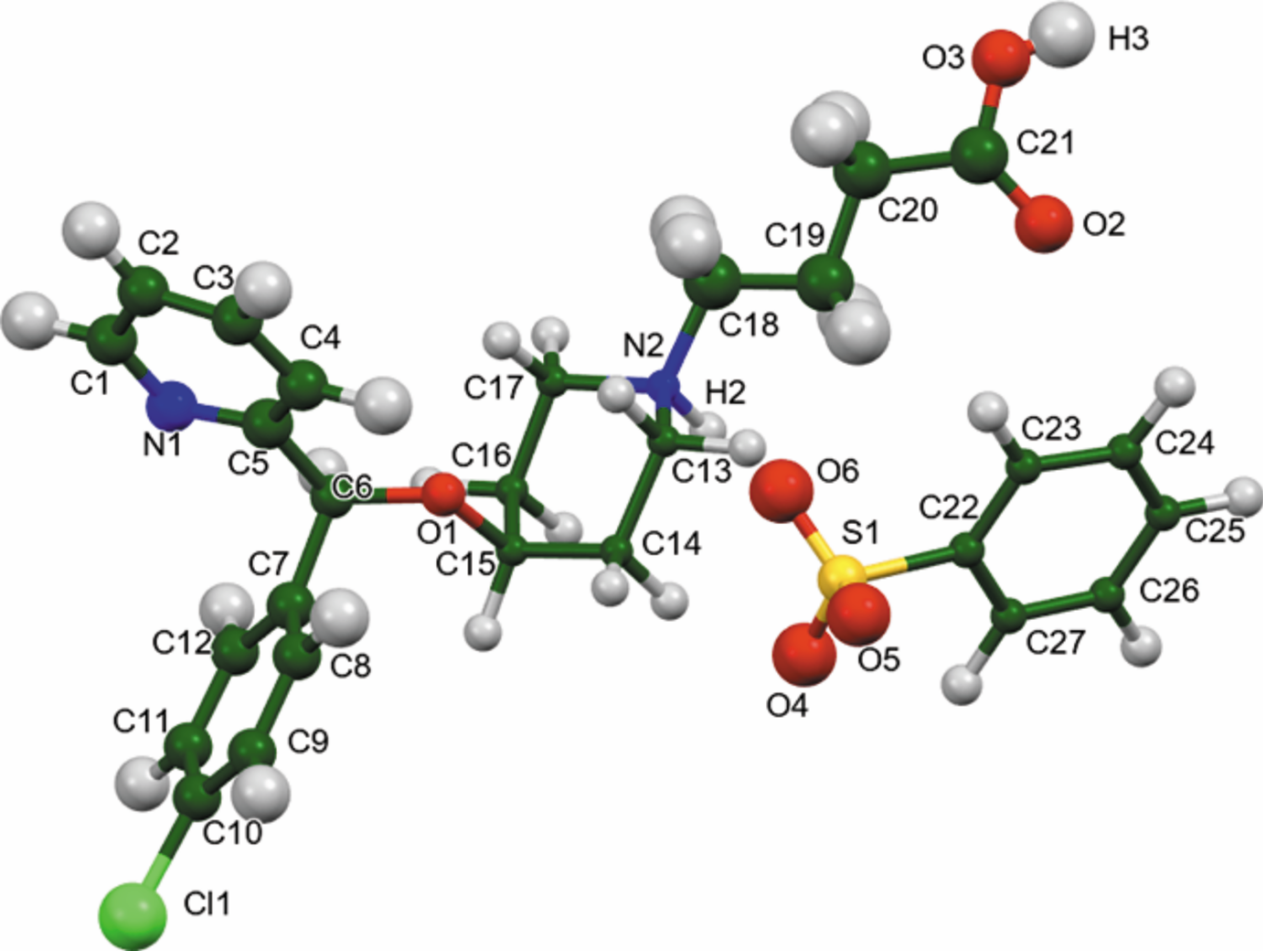
The asymmetric unit of bepotastine besylate, with the atom numbering. The atoms are represented by 50% probability spheroids/ellipsoids. Image generated using *Mercury* (Macrae *et al.*, 2020 ▸).

The r.m.s. Cartesian displacement between the recently-determined single-crystal (Wang *et al.*, 2025 ▸) and Rietveld-refined structures is 0.13 Å, the difference between the Rietveld-refined and *VASP*-optimized structures is 0.14 Å (*VASP* = Vienna Ab Initio Simulation Package; Kresse & Furthmüller, 1996 ▸). As expected, the model refined from single-crystal X-ray data and the model optimized with *VASP* are essentially identical. The Rietveld-refined model is almost as good. All of the differences are well within the normal range for correct structures (van de Streek & Neumann, 2014 ▸). The asymmetric unit with the atom numbering is presented in Fig. 1 ▸. The rest of this discussion will concentrate on the *VASP*-optimized structure.

All of the bond lengths, bond angles, and torsion angles fall within the normal ranges indicated by a *Mercury Mogul* Geometry check (Macrae *et al.*, 2020 ▸). Quantum chemical geometry optimization of the isolated cation (DFT/B3LYP/6-31G*/water) using *Spartan ’24* (Wavefunction, 2025 ▸) indicated that the solid-state conformation is 20.6 kJ mol^−1^ higher in energy than a local minimum. The global minimum-energy conformation is 208.2 kJ mol^−1^ lower in energy, but is much more com­pact (folded on itself). Inter­molecular inter­actions are thus important to determining conformation in the solid-state.

The isotropic displacement coefficients from this Rietveld refinement tend to be smaller than the equivalent *U*_iso_ calculated from the anisotropic coefficients of the single-crystal refinement. The difference may indicate an imperfect absorption model in the Rietveld refinement. The μ*R* was calculated using the tool on the 11-BM website (https://11b.x-ray.aps.anl.gov/absorb/), assuming a packing density of 50%. The packing density was not actually measured.

The availability of a structure refined from both single-crystal and powder data provides an opportunity to com­pare the precision (as well as the accuracy) of the two structures. The average standard uncertainties on the fractional coordinates are about three times larger in the structure refined from powder data than in the single-crystal one. The powder structure is thus accurate, but less precise than the single-crystal result.

**Figure 2:**
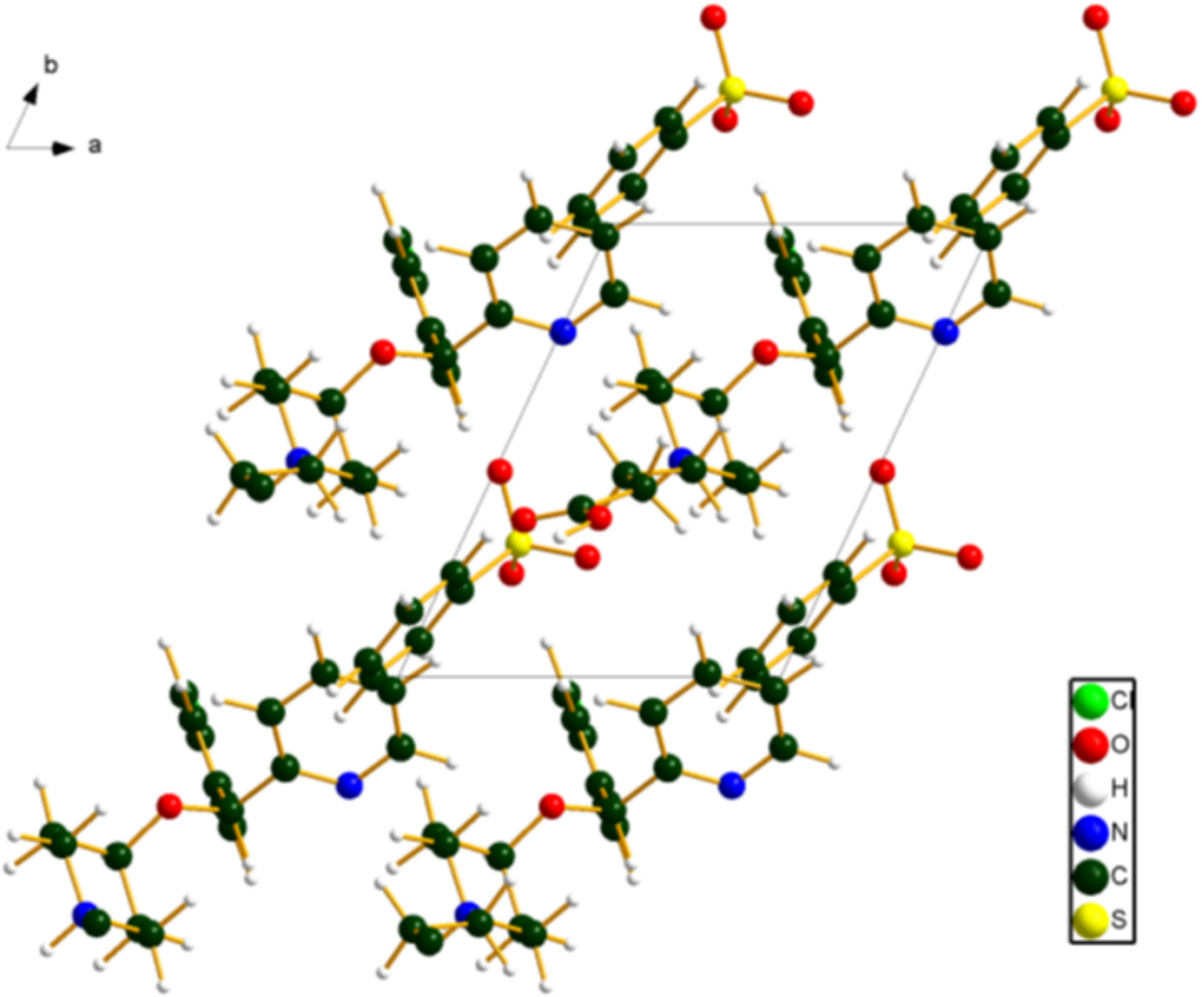
The crystal structure of bepotastine besylate, viewed down the *c* axis. Image generated using *DIAMOND* (Crystal Impact, 2025 ▸).

The crystal structure (Fig. 2 ▸) can be considered as layers parallel to the *bc* plane when viewed down the *b* axis, or as layers parallel to the (1
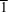
0) plane when viewed down the *c* axis. The *Mercury* Aromatics Analyser indicates one strong inter­action (4.87 Å) between the cation and anion. Other inter­actions are weak, with *d* > 8.02 Å.

Analysis of the contributions to the total crystal energy of the structure using the Forcite module of *Materials Studio* (Dassault Systèmes, 2024 ▸) suggests that the intra­molecular deformation energy is dominated by angle distortion terms, while van der Waals attractions (which in this force field-based analysis include hy­dro­gen bonds) dominate the inter­molecular energy.

There are two classical hy­dro­gen bonds in the crystal structure (Table 1 ▸). The cation makes a O3—H3⋯O4 hy­dro­gen bond to the anion, as well as an N2—H2⋯O6 one. These link the cations and anions into chains extending parallel to the *c* axis, with graph set (Etter, 1990 ▸; Bernstein *et al.*, 1995 ▸; Motherwell *et al.*, 2000 ▸) 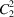
(11). The energy of the O—H⋯O hy­dro­gen bond was calculated using the correlation of Rammohan & Kaduk (2018 ▸), and the energy of the N—H⋯O hy­dro­gen bond was calculated using the correlation of Wheatley & Kaduk (2019 ▸). Several C—H⋯O/Cl/C hy­dro­gen bonds also contribute to the lattice energy (Table 1 ▸).

The Bravais–Friedel–Donnay–Harker (Bravais, 1866 ▸; Friedel, 1907 ▸; Donnay & Harker, 1937 ▸) morphology suggests that we might expect isotropic morphology for bepotastine besylate. A second-order spherical harmonic model was included in the refinement. The texture index was 1.006 (0), indicating that preferred orientation was not significant in this rotated capillary specimen.

## Synthesis and crystallization

Bepotastine besylate was a white powder purchased from TargetMol (Batch No. 132062), and was used as received.

## Refinement

Crystal data, data collection, and structure refinement details are summarized in Table 2 ▸. Reflections were indexed using *JADE Pro* (MDI, 2025 ▸) and the crystal structure was solved independently using direct methods as implemented in *EXPO2014* (Altomare *et al.*, 2013 ▸). Before refinement, we discovered the Wang *et al.* (2025 ▸) publication of this structure [it has not yet (as of March 2026) been added to the Cambridge Structural Database (Groom *et al.*, 2016 ▸)], and used their atom numbering.

**Figure 3:**
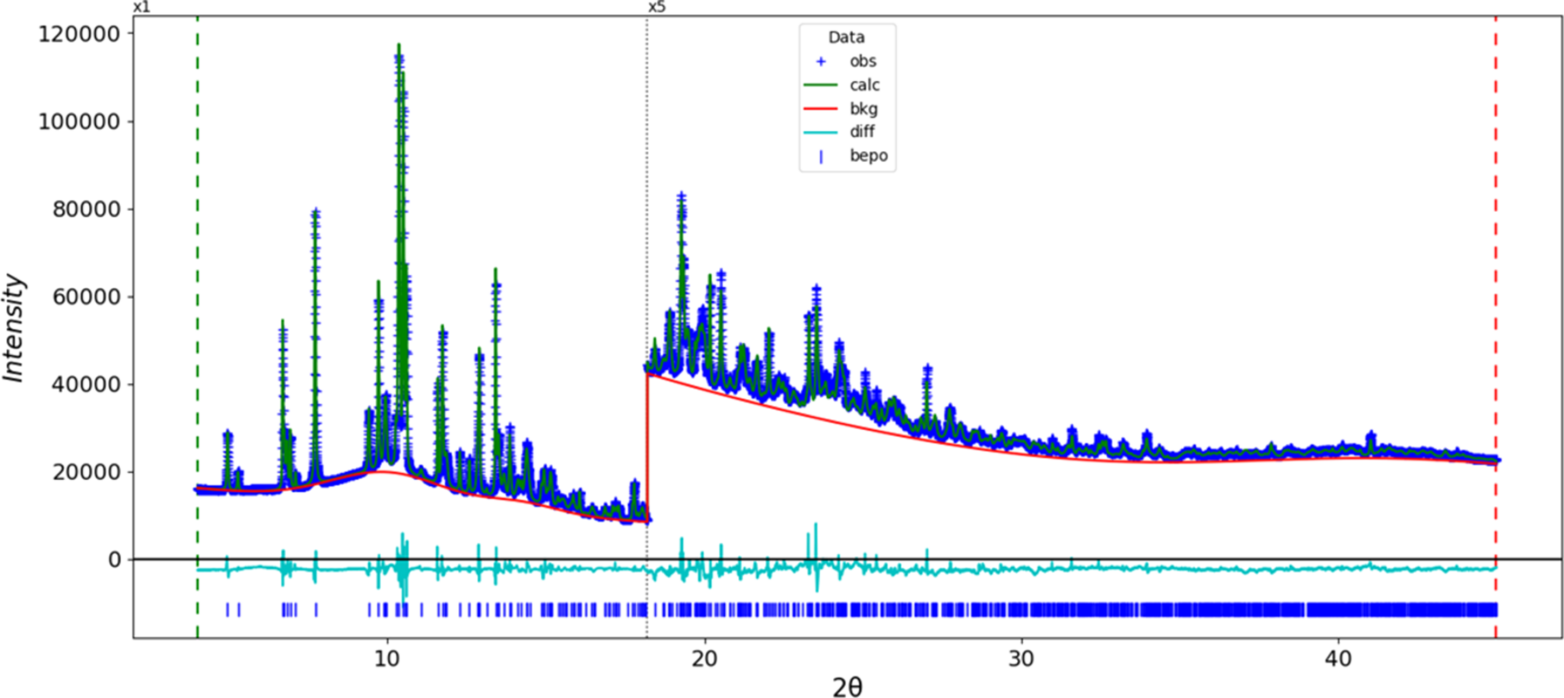
The Rietveld plot for bepotastine besylate. The blue crosses represent the observed data points, and the green line is the calculated pattern. The cyan curve is the normalized error plot, and the red line is the background curve. The blue tick marks indicate the peak positions. The vertical scale has been multiplied by a factor of ×5 for 2θ > 18.2°.

Rietveld refinement (Fig. 3 ▸) was carried out using *GSAS-II* (Toby & Von Dreele, 2013 ▸). All non-hy­dro­gen-bond lengths and angles were restrained according to a *Mercury*/*Mogul* Geometry Check (Sykes *et al.*, 2011 ▸; Bruno *et al.*, 2004 ▸). H atoms were included in calculated positions and recalculated during the refinement using the Adjust Hydrogen tool of *Materials Studio* (Dassault Systèmes, 2024 ▸). The coordinates of atom Cl1 were fixed to define the origin. *U*_iso_ of the C, N, and O atoms were grouped by chemical similarity, while the *U*_iso_ for H atoms were fixed at 1.3 times the *U*_iso_ of the C, N, and O atoms to which they are attached. Attempts to refine the Cl atom anisotropically led to an unreasonable ellipsoid, so it was refined isotropically. The final refinement yielded *R*_wp_ = 0.02700. The largest features in the normalized error plot are in the shapes of many of the strong low-angle reflections. In the difference-Fourier map, the residual maximum (1.98 Å from O2) and minimum (1.76 Å from C2) electron-density peaks were 0.16 (4) and −0.20 (4) e Å^−3^, respectively.

The crystal structure of bepotastine besylate was optimized (fixed Rietveld-refined unit cell) with density functional theory techniques using *VASP* (Version 6.0; Kresse & Furthmüller, 1996 ▸) through the *MedeA* graphical inter­face (Materials Design, 2024 ▸). Single-point density functional theory calculations (fixed experimental cell) and population analysis were carried out with *CRYSTAL23* (Erba *et al.*, 2023 ▸) using H, C, N, and O basis sets defined by Gatti *et al.* (1994 ▸) and the basis sets for S and Cl of Peintinger *et al.* (2013 ▸).

The powder pattern has been submitted to ICDD (Inter­national Centre for Diffraction Data) for inclusion in the Powder Diffraction File (PDF). The structure has been determined simultaneously using single-crystal techniques (Wang *et al.*, 2025 ▸).

## Supplementary Material

Crystal structure: contains datablock(s) I. DOI: 10.1107/S2414314626004244/zl4094sup1.cif

The VASP-optimized structure, with the hy­dro­gen bonds. DOI: 10.1107/S2414314626004244/zl4094sup2.txt

CCDC reference: 2548211

Additional supporting information:  crystallographic information; 3D view; checkCIF report

## Figures and Tables

**Table 1:** Hydrogen-bond geometry (Å, °)

*D*—H⋯*A*	*D*—H	H⋯*A*	*D*⋯*A*	*D*—H⋯*A*	Mulliken overlap	H-bond energy
O3—H3⋯O4	1.023	1.651	2.639	166.3		
N2—H2⋯O6	1.065	1.681	2.745	174.2		
						
*VASP*-optimized structure						
O3—H3⋯O4	1.023	1.651	2.639	166.3	0.067	14.0
N2—H2⋯O6	1.065	1.681	2.745	174.2	0.075	6.3
C2—H2*A*⋯O4	1.091	2.814	3.850	157.4	0.010	
C3—H3*A*⋯O6	1.098	2.581	3.341	127.8	0.012	
C4—H4⋯O1	1.101	2.471	2.786	95.1	0.010	
C6—H2⋯O2	1.107	2.491	3.570	170.8	0.013	
C8—H8⋯O4	1.102	2.881	3.903	161.0	0.010	
C9—H9⋯O2	1.092	2.489	3.517	158.7	0.019	
C11—H11⋯O5	1.100	2.188	3.176	152.8	0.034	
C13—H13*A*⋯Cl1	1.098	3.044	4.137	170.2	0.013	
C14—H14*B*⋯O5	1.099	2.568	3.461	139.4	0.014	
C15—H15⋯C7	1.100	2.681	3.045	99.2	0.014	
C16—H16*A*⋯O3	1.100	2.612	3.534	139.4	0.011	
C17—H17*A*⋯O1	1.098	2.675	2.972	93.9	0.010	

**Table 2:** Experimental details

Crystal data	
Chemical formula	C_21_H_26_ClN_2_O_3_^+^·C_6_H_5_O_3_S^−^
*M*_r_	547.07
Crystal system, space group	Triclinic, *P*1
Temperature (K)	298
*a*, *b*, *c* (Å)	8.0153 (6), 9.8211 (5), 10.2345 (10)
α, β, γ (°)	88.1641 (17), 68.962 (2), 65.8917 (8)
*V* (Å^3^)	680.12 (2)
*Z*	1
Radiation type	Synchrotron, λ = 0.81933 Å
Specimen shape, size (mm)	Cylinder, 0.45 × 0.15

Data collection	
Diffractometer	Wiggler Low-Energy Beamline, Brockhouse X-ray Diffraction and Scattering Sector, Canadian Light Source
Specimen mounting	Kapton capillary
Data collection mode	Transmission
Scan method	Step
2θ values (°)	2θ_min_ = −9.008 2θ_max_ = 75.047, 2θ_step_ = 0.003

Refinement	
*R* factors and goodness of fit	*R*_p_ = 0.018, *R*_wp_ = 0.027, *R*_exp_ = 0.002, *R*(*F*^2^) = 0.04412, χ^2^ = 125.956
No. of parameters	157
No. of restraints	94
(Δ/σ)_max_	2.333

Computer program: *GSAS-II* (Toby & Von Dreele, 2013 ▸).

## full crystallographic data

### 1-(3-Carboxypropyl)-4-[(4-chlorophenyl)(pyridin-2-yl)methoxy]piperidin-1-ium benzenesulfonate Crystal data

**Table:**

C_21_H_26_ClN_2_O_3_^+^·C_6_H_5_O_3_S^−^	γ = 65.8917 (8)°
*M**_r_* = 547.07	*V* = 680.12 (2) Å^3^
Triclinic, *P*1	*Z* = 1
*a* = 8.0153 (6) Å	*D*_x_ = 1.336 Mg m^−^^3^
*b* = 9.8211 (5) Å	Synchrotron radiation, λ = 0.81933 Å
*c* = 10.2345 (10) Å	*T* = 298 K
α = 88.1641 (17)°	cylinder, 0.45 × 0.15 mm
β = 68.962 (2)°	

### 1-(3-Carboxypropyl)-4-[(4-chlorophenyl)(pyridin-2-yl)methoxy]piperidin-1-ium benzenesulfonate Data collection

**Table:**

Wiggler Low-Energy Beamline, Brockhouse X-ray Diffraction and Scattering Sector, Canadian Light Source diffractometer	Scan method: step
Specimen mounting: Kapton capillary	2θ_min_ = −9.008°, 2θ_max_ = 75.047°, 2θ_step_ = 0.003°
Data collection mode: transmission	

### 1-(3-Carboxypropyl)-4-[(4-chlorophenyl)(pyridin-2-yl)methoxy]piperidin-1-ium benzenesulfonate Refinement

**Table:**

Least-squares matrix: full	157 parameters
*R*_p_ = 0.018	94 restraints
*R*_wp_ = 0.027	28 constraints
*R*_exp_ = 0.002	Weighting scheme based on measured s.u.'s
*R*(*F*^2^) = 0.04412	(Δ/σ)_max_ = 2.333
33623 data points	Background function: Background function: "chebyschev-1" function with 3 terms: 7.82(4)e3, -6.61(6)e3, 1.72(3)e3, Background peak parameters: pos, int, sig, gam: 10.042(8), 3.336(18)e6, 3.30(4)e4, 0.100, 14.208(20), 7.15(14)e5, 1.43(4)e4, 0.100, 41.97(20), 2.36(23)e6, 3.42(23)e5, 0.100,
Profile function: Finger-Cox-Jephcoat function parameters U, V, W, X, Y, SH/L: peak variance(Gauss) = Utan(Th)^2^+Vtan(Th)+W: peak HW(Lorentz) = X/cos(Th)+Ytan(Th); SH/L = S/L+H/L U, V, W in (centideg)^2^, X & Y in centideg 6.157, -1.198, 1.258, 0.000, 0.667, 0.002,	Preferred orientation correction: Simple spherical harmonic correction Order = 2 Coefficients: 0:0:C(2,-2) = 0.042(5); 0:0:C(2,-1) = 0.110(6); 0:0:C(2,0) = 0.009(7); 0:0:C(2,1) = -0.134(5); 0:0:C(2,2) = 0.031(5)

### 1-(3-Carboxypropyl)-4-[(4-chlorophenyl)(pyridin-2-yl)methoxy]piperidin-1-ium benzenesulfonate Fractional atomic coordinates and isotropic or equivalent isotropic displacement parameters (Å^2^)

**Table:**

	*x*	*y*	*z*	*U*_iso_*/*U*_eq_	
Cl1	0.50540	0.93250	−0.60523	0.082 (3)*	
O1	0.5505 (16)	0.7147 (12)	0.0188 (10)	0.034 (4)*	
O2	0.116 (2)	0.3636 (16)	0.7826 (12)	0.059 (3)*	
O3	0.3338 (16)	0.3491 (14)	0.8727 (12)	0.059 (3)*	
H3	0.29300	0.30400	0.94400	0.0778*	
N1	0.9969 (14)	0.7676 (10)	−0.1821 (9)	0.046 (3)*	
N2	0.4631 (16)	0.4856 (11)	0.2721 (11)	0.020 (3)*	
H2	0.40680	0.40890	0.25650	0.0255*	
C1	1.0962 (17)	0.8438 (16)	−0.1643 (13)	0.046 (3)*	
H1	1.25014	0.81896	−0.24501	0.0595*	
C2	1.0220 (19)	0.9541 (17)	−0.0509 (15)	0.046 (3)*	
H2A	1.11912	1.00663	−0.03757	0.0595*	
C3	0.832 (2)	0.9940 (16)	0.0402 (12)	0.046 (3)*	
H3A	0.75694	1.09371	0.12692	0.0595*	
C4	0.7301 (16)	0.9122 (16)	0.0268 (13)	0.046 (3)*	
H4	0.57426	0.94103	0.10643	0.0595*	
C5	0.8204 (13)	0.7980 (13)	−0.0828 (12)	0.046 (3)*	
C6	0.7188 (15)	0.7061 (11)	−0.1060 (9)	0.034 (4)*	
H6	0.83146	0.58331	−0.13717	0.0444*	
C7	0.6533 (17)	0.7563 (11)	−0.2276 (9)	0.041 (3)*	
C8	0.510 (2)	0.8993 (12)	−0.2153 (9)	0.041 (3)*	
H8	0.43155	0.97627	−0.11032	0.0624*	
C9	0.4590 (19)	0.9524 (10)	−0.3287 (10)	0.041 (3)*	
H9	0.33604	1.06955	−0.31503	0.0624*	
C10	0.559 (2)	0.8612 (11)	−0.4575 (8)	0.041 (3)*	
C11	0.7068 (18)	0.7186 (10)	−0.4722 (9)	0.041 (3)*	
H11	0.78915	0.64441	−0.57890	0.0534*	
C12	0.7522 (18)	0.6682 (10)	−0.3570 (10)	0.041 (3)*	
H12	0.87456	0.55082	−0.37036	0.0534*	
C13	0.3185 (17)	0.6382 (13)	0.2781 (11)	0.020 (3)*	
H13A	0.37653	0.72188	0.29482	0.0255*	
H13B	0.17356	0.66131	0.37000	0.0255*	
C14	0.2792 (16)	0.6589 (15)	0.1392 (14)	0.020 (3)*	
H14A	0.17877	0.78213	0.14286	0.0253*	
H14B	0.20237	0.58660	0.12936	0.0255*	
C15	0.4635 (18)	0.6143 (15)	0.0121 (11)	0.020 (3)*	
H15	0.43091	0.62040	−0.08812	0.0255*	
C16	0.6116 (17)	0.4627 (13)	0.0115 (12)	0.020 (3)*	
H16A	0.55409	0.37582	0.00356	0.0255*	
H16B	0.75479	0.43672	−0.08318	0.0255*	
C17	0.6513 (15)	0.4550 (13)	0.1465 (13)	0.020 (3)*	
H17A	0.70654	0.54294	0.15589	0.0255*	
H17B	0.76819	0.33815	0.14360	0.0255*	
C18	0.495 (2)	0.4649 (17)	0.4089 (13)	0.059 (3)*	
H18A	0.49294	0.57254	0.44961	0.0773*	
H18B	0.64553	0.36826	0.38954	0.0773*	
C19	0.349 (2)	0.430 (2)	0.5176 (14)	0.059 (3)*	
H19A	0.35115	0.32131	0.47972	0.0773*	
H19B	0.19654	0.52637	0.54307	0.0773*	
C20	0.404 (2)	0.410 (2)	0.6477 (14)	0.059 (3)*	
H20A	0.54754	0.30528	0.62425	0.0773*	
H20B	0.42765	0.51206	0.67129	0.0773*	
C21	0.2547 (19)	0.3924 (17)	0.7812 (13)	0.059 (3)*	
S1	0.1428 (11)	0.3047 (8)	0.2397 (8)	0.043 (3)*	
O4	0.1779 (16)	0.2340 (12)	0.1101 (12)	0.072 (3)*	
O5	−0.0095 (17)	0.4590 (12)	0.2794 (12)	0.072 (3)*	
O6	0.3278 (18)	0.2767 (14)	0.2400 (13)	0.072 (3)*	
C22	0.053 (2)	0.1955 (14)	0.3627 (9)	0.023 (3)*	
C23	0.028 (2)	0.2166 (15)	0.5031 (11)	0.023 (3)*	
H23	0.07729	0.29738	0.53849	0.0299*	
C24	−0.059 (2)	0.1374 (15)	0.5982 (10)	0.023 (3)*	
H24	−0.09068	0.15850	0.71538	0.0299*	
C25	−0.106 (2)	0.0357 (16)	0.5530 (12)	0.023 (3)*	
H25	−0.16802	−0.03343	0.63058	0.0299*	
C26	−0.079 (3)	0.0132 (16)	0.4144 (14)	0.023 (3)*	
H26	−0.12166	−0.07253	0.37851	0.0299*	
C27	−0.002 (2)	0.0933 (16)	0.3172 (11)	0.023 (3)*	
H27	0.01667	0.07747	0.20196	0.0299*	

### 1-(3-Carboxypropyl)-4-[(4-chlorophenyl)(pyridin-2-yl)methoxy]piperidin-1-ium benzenesulfonate Geometric parameters (Å, º)

**Table:**

Cl1—C10	1.765 (6)	H14A—C14	1.140 (12)
O1—C6	1.461 (8)	H14B—C14	1.140 (12)
O1—C15	1.437 (5)	C15—O1	1.437 (5)
O2—C21	1.247 (8)	C15—C14	1.481 (8)
O3—H3	0.873 (9)	C15—H15	1.141 (9)
O3—C21	1.281 (9)	C15—C16	1.476 (8)
H3—O3	0.873 (9)	H15—C15	1.141 (9)
N1—C1	1.352 (6)	C16—C15	1.476 (8)
N1—C5	1.329 (5)	C16—H16A	1.140 (11)
N2—H2	1.065 (9)	C16—H16B	1.140 (10)
N2—C13	1.460 (7)	C16—C17	1.518 (8)
N2—C17	1.510 (7)	H16A—C16	1.140 (11)
N2—C18	1.504 (8)	H16B—C16	1.140 (10)
H2—N2	1.065 (9)	C17—N2	1.510 (7)
C1—N1	1.352 (6)	C17—C16	1.518 (8)
C1—H1	1.140 (9)	C17—H17A	1.140 (12)
C1—C2	1.391 (8)	C17—H17B	1.140 (10)
H1—C1	1.140 (9)	H17A—C17	1.140 (12)
C2—C1	1.391 (8)	H17B—C17	1.140 (10)
C2—H2A	1.140 (10)	C18—N2	1.504 (8)
C2—C3	1.363 (7)	C18—H18A	1.140 (14)
H2A—C2	1.140 (10)	C18—H18B	1.140 (14)
C3—C2	1.363 (7)	C18—C19	1.446 (10)
C3—H3A	1.140 (8)	H18A—C18	1.140 (14)
C3—C4	1.399 (6)	H18B—C18	1.140 (14)
H3A—C3	1.140 (8)	C19—C18	1.446 (10)
C4—C3	1.399 (6)	C19—H19A	1.140 (18)
C4—H4	1.140 (8)	C19—H19B	1.140 (19)
C4—C5	1.369 (6)	C19—C20	1.527 (10)
H4—C4	1.140 (8)	H19A—C19	1.140 (18)
C5—N1	1.329 (5)	H19B—C19	1.140 (19)
C5—C4	1.369 (6)	C20—C19	1.527 (10)
C5—C6	1.512 (4)	C20—H20A	1.140 (18)
C6—O1	1.461 (8)	C20—H20B	1.140 (17)
C6—C5	1.512 (4)	C20—C21	1.519 (7)
C6—H6	1.140 (10)	H20A—C20	1.140 (18)
C6—C7	1.512 (5)	H20B—C20	1.140 (17)
H6—C6	1.140 (10)	C21—O2	1.247 (8)
C7—C6	1.512 (5)	C21—O3	1.281 (9)
C7—C8	1.377 (5)	C21—C20	1.519 (7)
C7—C12	1.375 (5)	S1—O4	1.397 (9)
C8—C7	1.377 (5)	S1—O5	1.458 (8)
C8—H8	1.139 (8)	S1—O6	1.394 (9)
C8—C9	1.384 (6)	S1—C22	1.774 (5)
H8—C8	1.139 (8)	O4—S1	1.397 (9)
C9—C8	1.384 (6)	O5—S1	1.458 (8)
C9—H9	1.140 (8)	O6—S1	1.394 (9)
C9—C10	1.383 (6)	C22—S1	1.774 (5)
H9—C9	1.140 (8)	C22—C23	1.388 (5)
C10—Cl1	1.765 (6)	C22—C27	1.401 (5)
C10—C9	1.383 (6)	C23—C22	1.388 (5)
C10—C11	1.384 (6)	C23—H23	1.140 (9)
C11—C10	1.384 (6)	C23—C24	1.392 (6)
C11—H11	1.140 (8)	H23—C23	1.140 (9)
C11—C12	1.375 (5)	C24—C23	1.392 (6)
H11—C11	1.140 (8)	C24—H24	1.140 (10)
C12—C7	1.375 (5)	C24—C25	1.346 (7)
C12—C11	1.375 (5)	H24—C24	1.140 (10)
C12—H12	1.140 (7)	C25—C24	1.346 (7)
H12—C12	1.140 (7)	C25—H25	1.140 (9)
C13—N2	1.460 (7)	C25—C26	1.369 (8)
C13—H13A	1.140 (12)	H25—C25	1.140 (9)
C13—H13B	1.140 (11)	C26—C25	1.369 (8)
C13—C14	1.552 (8)	C26—H26	1.140 (10)
H13A—C13	1.140 (12)	C26—C27	1.377 (6)
H13B—C13	1.140 (11)	H26—C26	1.140 (10)
C14—C13	1.552 (8)	C27—C22	1.401 (5)
C14—H14A	1.140 (12)	C27—C26	1.377 (6)
C14—H14B	1.140 (12)	C27—H27	1.141 (9)
C14—C15	1.481 (8)	H27—C27	1.141 (9)
			
C6—O1—C15	117.3 (7)	C14—C15—H15	110.6 (9)
H3—O3—C21	124.8 (10)	O1—C15—C16	105.4 (6)
C1—N1—C5	117.5 (3)	C14—C15—C16	111.7 (5)
H2—N2—C13	108.3 (9)	H15—C15—C16	110.6 (10)
H2—N2—C17	108.2 (8)	C15—C16—H16A	109.4 (10)
C13—N2—C17	108.7 (5)	C15—C16—H16B	109.3 (10)
H2—N2—C18	108.3 (7)	H16A—C16—H16B	109.3 (8)
C13—N2—C18	110.6 (7)	C15—C16—C17	110.2 (4)
C17—N2—C18	112.6 (6)	H16A—C16—C17	109.1 (10)
N1—C1—H1	120.0 (8)	H16B—C16—C17	109.4 (10)
N1—C1—C2	123.8 (4)	N2—C17—C16	109.6 (5)
H1—C1—C2	116.2 (9)	N2—C17—H17A	109.5 (9)
C1—C2—H2A	120.0 (9)	C16—C17—H17A	109.4 (8)
C1—C2—C3	116.9 (5)	N2—C17—H17B	109.5 (9)
H2A—C2—C3	123.1 (10)	C16—C17—H17B	109.4 (10)
C2—C3—H3A	120.0 (10)	H17A—C17—H17B	109.5 (8)
C2—C3—C4	119.7 (4)	N2—C18—H18A	108.7 (10)
H3A—C3—C4	120.3 (9)	N2—C18—H18B	109.5 (9)
C3—C4—H4	120.0 (8)	H18A—C18—H18B	108.7 (10)
C3—C4—C5	119.0 (3)	N2—C18—C19	113.9 (7)
H4—C4—C5	121.0 (8)	H18A—C18—C19	108.7 (14)
N1—C5—C4	122.5 (3)	H18B—C18—C19	107.3 (12)
N1—C5—C6	115.2 (4)	C18—C19—H19A	109.8 (14)
C4—C5—C6	122.2 (4)	C18—C19—H19B	109.5 (14)
O1—C6—C5	113.8 (5)	H19A—C19—H19B	109.8 (10)
O1—C6—H6	108.0 (7)	C18—C19—C20	107.5 (6)
C5—C6—H6	108.0 (7)	H19A—C19—C20	109.8 (15)
O1—C6—C7	109.9 (6)	H19B—C19—C20	110.5 (15)
C5—C6—C7	109.1 (5)	C19—C20—H20A	109.5 (16)
H6—C6—C7	107.9 (5)	C19—C20—H20B	108.3 (13)
C6—C7—C8	120.0 (4)	H20A—C20—H20B	108.3 (10)
C6—C7—C12	120.6 (4)	C19—C20—C21	116.2 (6)
C8—C7—C12	118.9 (3)	H20A—C20—C21	106.0 (11)
C7—C8—H8	120.0 (6)	H20B—C20—C21	108.3 (12)
C7—C8—C9	121.1 (3)	O2—C21—O3	121.3 (8)
H8—C8—C9	118.9 (6)	O2—C21—C20	123.6 (8)
C8—C9—H9	120.0 (6)	O3—C21—C20	109.4 (8)
C8—C9—C10	118.9 (3)	O4—S1—O5	115.2 (7)
H9—C9—C10	121.1 (6)	O4—S1—O6	106.2 (7)
Cl1—C10—C9	119.2 (4)	O5—S1—O6	118.2 (7)
Cl1—C10—C11	120.3 (4)	O4—S1—C22	103.0 (4)
C9—C10—C11	120.5 (3)	O5—S1—C22	107.0 (5)
C10—C11—H11	120.0 (5)	O6—S1—C22	106.0 (5)
C10—C11—C12	119.3 (3)	S1—C22—C23	120.7 (4)
H11—C11—C12	120.8 (6)	S1—C22—C27	118.7 (4)
C7—C12—C11	121.3 (3)	C23—C22—C27	120.5 (3)
C7—C12—H12	120.0 (6)	C22—C23—H23	119.9 (7)
C11—C12—H12	118.7 (6)	C22—C23—C24	118.7 (3)
N2—C13—H13A	109.2 (9)	H23—C23—C24	121.4 (7)
N2—C13—H13B	109.4 (9)	C23—C24—H24	120.0 (8)
H13A—C13—H13B	109.2 (9)	C23—C24—C25	120.5 (4)
N2—C13—C14	110.9 (5)	H24—C24—C25	119.4 (8)
H13A—C13—C14	109.3 (10)	C24—C25—H25	120.0 (9)
H13B—C13—C14	108.7 (9)	C24—C25—C26	121.1 (4)
C13—C14—H14A	109.0 (11)	H25—C25—C26	118.9 (9)
C13—C14—H14B	109.5 (10)	C25—C26—H26	120.0 (9)
H14A—C14—H14B	108.9 (8)	C25—C26—C27	120.6 (4)
C13—C14—C15	112.5 (4)	H26—C26—C27	119.4 (8)
H14A—C14—C15	109.0 (9)	C22—C27—C26	118.5 (3)
H14B—C14—C15	108.0 (10)	C22—C27—H27	120.3 (7)
O1—C15—C14	107.7 (6)	C26—C27—H27	121.2 (7)
O1—C15—H15	110.6 (9)		

### 1-(3-Carboxypropyl)-4-[(4-chlorophenyl)(pyridin-2-yl)methoxy]piperidin-1-ium benzenesulfonate Hydrogen-bond geometry (Å, º)

**Table:**

*D*—H···*A*	*D*—H	H···*A*	*D*···*A*	*D*—H···*A*
O3—H3···O4	1.023	1.651	2.639	166.3
N2—H2···O6	1.065	1.681	2.745	174.2
